# Time-robust myocardial [^68^Ga]Ga-FAPI PET biomarker reflects aortic stenosis severity and predicts post-TAVI outcomes

**DOI:** 10.1007/s00259-026-07815-4

**Published:** 2026-02-16

**Authors:** Song Xue, Qianling Ye, Aleksa Lazarević, Kevin Hamzaraj, Patrick Binder, Christian Nitsche, Attila Kiss, Bruno K Podesser, Marcus Hacker, Xiang Li, Raffaella Calabretta

**Affiliations:** 1 Division of Nuclear Medicine, Department of Biomedical Imaging and Image-guided Therapy, Vienna General Hospital, Medical University of Vienna, Vienna, Austria; 2 Ludwig Boltzmann Institute for Cardiovascular Research, Center for Biomedical Research and Translational Surgery, Medical University of Vienna, Vienna, Austria; 3 Division of Cardiology, Department of Internal Medicine II, Medical University of Vienna, Vienna, Austria; 4 Department of Nuclear Medicine, Beijing Chest Hospital, Capital Medical University, Beijing, China

**Keywords:** Aortic stenosis, TAVI, [^68^Ga]Ga-FAPI PET, Myocardial fibrosis, Imaging biomarker

## Abstract

**Background:**

Aortic stenosis (AS) induces myocardial remodeling and fibroblast activation, yet modifiable biomarkers capable of capturing active fibrogenesis and predicting post-transcatheter aortic valve implantation (TAVI) recovery are currently scarce. Fibroblast activation protein (FAP)–targeted PET serves as a noninvasive tool to visualize activated fibroblasts in vivo. We evaluated a time-robust, blood-pool–normalized myocardial [^68^Ga]Ga-FAPI PET imaging biomarker that reflects AS burden and predicts outcomes after TAVI.

**Methods:**

Nineteen patients with severe symptomatic AS underwent [^68^Ga]Ga-FAPI-04 PET/CT at 60, 70, and 120 min. Using an in-house semi-automatic pipeline, the left ventricular (LV) myocardium was segmented, and regions of elevated fibroblast activity (EFM) were delineated using a blood-pool–anchored, time-point–specific threshold. We quantified myocardial SUV_mean_, blood-pool SUV_mean_, and a normalized myocardium-to-blood index, TBR(EFM), and assessed associations with N-terminal pro-brain natriuretic peptide (NT-proBNP) and left-ventricular ejection fraction (LVEF). One-year outcomes (*n* = 11) were assessed using a predefined composite clinical response.

**Results:**

Blood-pool SUV_mean_ declined from 60 to 120 min, whereas myocardial SUV_mean_ decreased less, yielding stable TBR(EFM) across time points (60/70/120 min: 2.2 ± 0.8, 2.1 ± 0.9, 2.3 ± 0.9; ANOVA *p* = 0.596). By contrast, myocardial SUV_mean_ fell from 3.8 ± 0.7 (60 min) to 2.1 ± 0.9 (120 min; *p* < 0.001). TBR(EFM) correlated with NT-proBNP at all time-points (60 min *r* = 0.65, *p* = 0.007; 120 min *r* = 0.72, *p* = 0.003), whereas SUV_mean_ did not (60 min *p* = 0.576; 120 min *p* = 0.109). Baseline TBR(EFM) was significantly lower in one-year responders than non-responders (1.7 ± 0.2 vs. 2.9 ± 0.9; *p* = 0.013), with separation present at each time point (*p* < 0.05). Higher baseline TBR(EFM) associated with lower reductions in NT-proBNP at one year (*p* < 0.05).

**Conclusions:**

Myocardial [^68^Ga]Ga-FAPI TBR may provide a time-robust index of active fibroblast signaling that relates to myocardial hemodynamic stress and stratifies one-year clinical response after TAVI. A single 60-minute acquisition with TBR quantification may be sufficient for myocardial [^68^Ga]Ga-FAPI assessment. These hypothesis-generating findings require validation in larger, multicenter cohorts.

**Graphical abstract:**

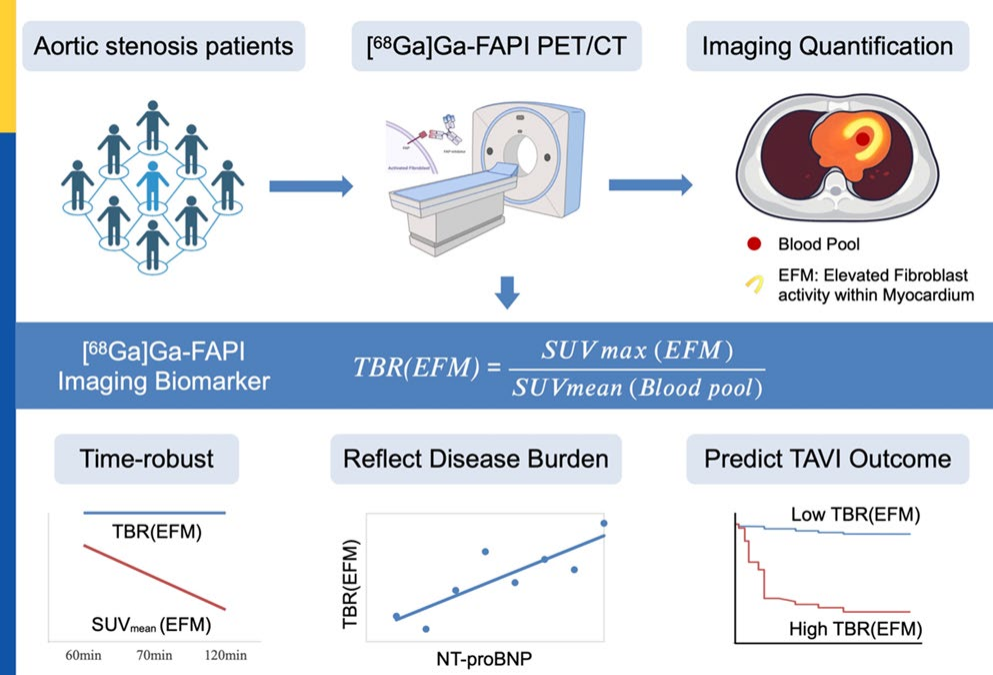

**Supplementary Information:**

The online version contains supplementary material available at 10.1007/s00259-026-07815-4.

## Introduction

Severe aortic stenosis (AS) is a progressive disease characterized not only by valvular obstruction but also by maladaptive left-ventricular (LV) remodeling, which is associated with hypertrophy and fibrosis, resulting from fibroblast-to-myofibroblast transition and accelerated interstitial matrix turnover [1]. While aortic valve replacement, namely surgical or transcatheter aortic valve implantation (TAVI), relieves afterload, mitigates symptoms and improves long-term survival for the majority of AS patients, responses are heterogeneous. Approximately 20% of patients are hospitalized shortly after discharge, and nearly 25% experience at least one readmission within the first year following TAVI [2, 3]. A substantial subset develops persistent heart failure, adverse remodeling, or limited functional recovery despite technically successful interventions [4, 5]. Identifying patients with active myocardial fibrogenesis—distinct from accumulated scar—could refine timing of intervention, enable risk stratification, and guide adjunctive medical therapy beyond valve replacement. Conventional echocardiography captures hemodynamic severity and global function, and cardiac MRI (late gadolinium enhancement, native T1, extracellular volume) quantifies fibrosis burden; however, these techniques primarily index structural end-products rather than ongoing and active process of fibrosis and may not fully capture dynamic, potentially reversible processes in the peri-procedural window.

Fibroblast activation protein (FAP), a cell-surface serine protease upregulated on activated fibroblasts but minimally expressed in quiescent fibroblasts and normal myocardium, has emerged as a compelling target for molecular imaging [6, 7]. Radiolabeled FAP inhibitors (FAPI), such [^68^Ga]Ga-FAPI, enable noninvasive positron emission tomography (PET) imaging of fibroblast activation across organs. Increased myocardial [^68^Ga]Ga-FAPI uptake has been demonstrated in conditions characterized by active fibrotic remodeling, including recent myocardial infarction, myocarditis, hypertensive heart disease, cardiac sarcoidosis, and pressure overload [8, 9]. Early studies in AS reported elevated pre-TAVI myocardial FAPI signal compared with control cohorts without cardiac disease, along with associations to natriuretic peptides and functional measures [10], suggesting that [^68^Ga]Ga-FAPI PET may inform risk stratification and recovery potential. In contrast to [^18^F]FDG, which is confounded by variable cardiomyocyte glucose utilization, the [^68^Ga]Ga-FAPI signal reflects stromal biology and can be interpreted without stringent dietary preparation [11]. Thus, [^68^Ga]Ga-FAPI-PET offers a mechanistically specific window into active fibrogenesis that complements structural MRI markers and functional echocardiography.

A principal obstacle to the broader clinical adoption of myocardial [^68^Ga]Ga-FAPI imaging is the lack of standardized quantification protocols. Optimal acquisition timing and reliable methods for assessing myocardial FAPI expression remain undefined, and the prognostic relevance of pre-TAVI FAPI uptake for post-TAVI outcomes is not yet established. This study aimed to (i) identify a robust imaging window and reproducible quantitative metric for myocardial FAPI assessment, (ii) characterize the association between baseline myocardial FAPI uptake and circulating N-terminal pro-brain natriuretic peptide (NT-proBNP) as a biomarker of hemodynamic stress, and (iii) determine whether pre-TAVI myocardial FAPI signal predicts clinical outcomes one year after TAVI. We hypothesized that the myocardium-to-blood pool ratio TBR(EFM) would serve as a time-robust and clinically meaningful indicator of fibroblast activity, superior to raw SUV_mean_, and that elevated baseline TBR would predispose a substantial attenuation of myocardial reverse remodeling.

## Materials and methods

### Patient population

This study included 19 patients with severe, symptomatic AS who were scheduled for TAVI and underwent [^68^Ga]Ga-FAPI-04 PET/CT at the Division of Nuclear Medicine, Medical University of Vienna, as part of clinical studies (Approval Nr. 1971/2023 and Approval Nr. 1400/2000). All participants provided written informed consent, and the study was approved by the institutional ethics committee in accordance with the Declaration of Helsinki.

### Imaging protocol

All participants underwent PET/CT with an in-house–synthesized, GMP-compliant [^68^Ga]Ga-FAPI-04 tracer within an average of 2 months (range, 0.2–5.5 months) before TAVI. Whole-body [^68^Ga]Ga-FAPI-04 PET/CT was performed approximately 60 min post-injection (p.i.) following intravenous administration of ~ 180 MBq of tracer, covering the vertex to the proximal femurs. Additional static cardiac PET/CT acquisitions were performed at 70 and 120 min p.i.

All scans were acquired on a Siemens Biograph TruePoint 64 PET/CT system (Siemens Healthineers, Erlangen, Germany). A low-dose CT without intravenous contrast was acquired for attenuation correction and anatomic localization (120 kV; 200–230 mAs; slice thickness, 3 mm; increment, 2 mm). CT acquisition was performed during shallow breathing to match PET and CT slices. PET acquisition time was 3–4 min per bed position. PET and CT images were co-registered and fused into 5-mm-thick transaxial and coronal images.

### Image processing and quantification

**Fig. 1:**
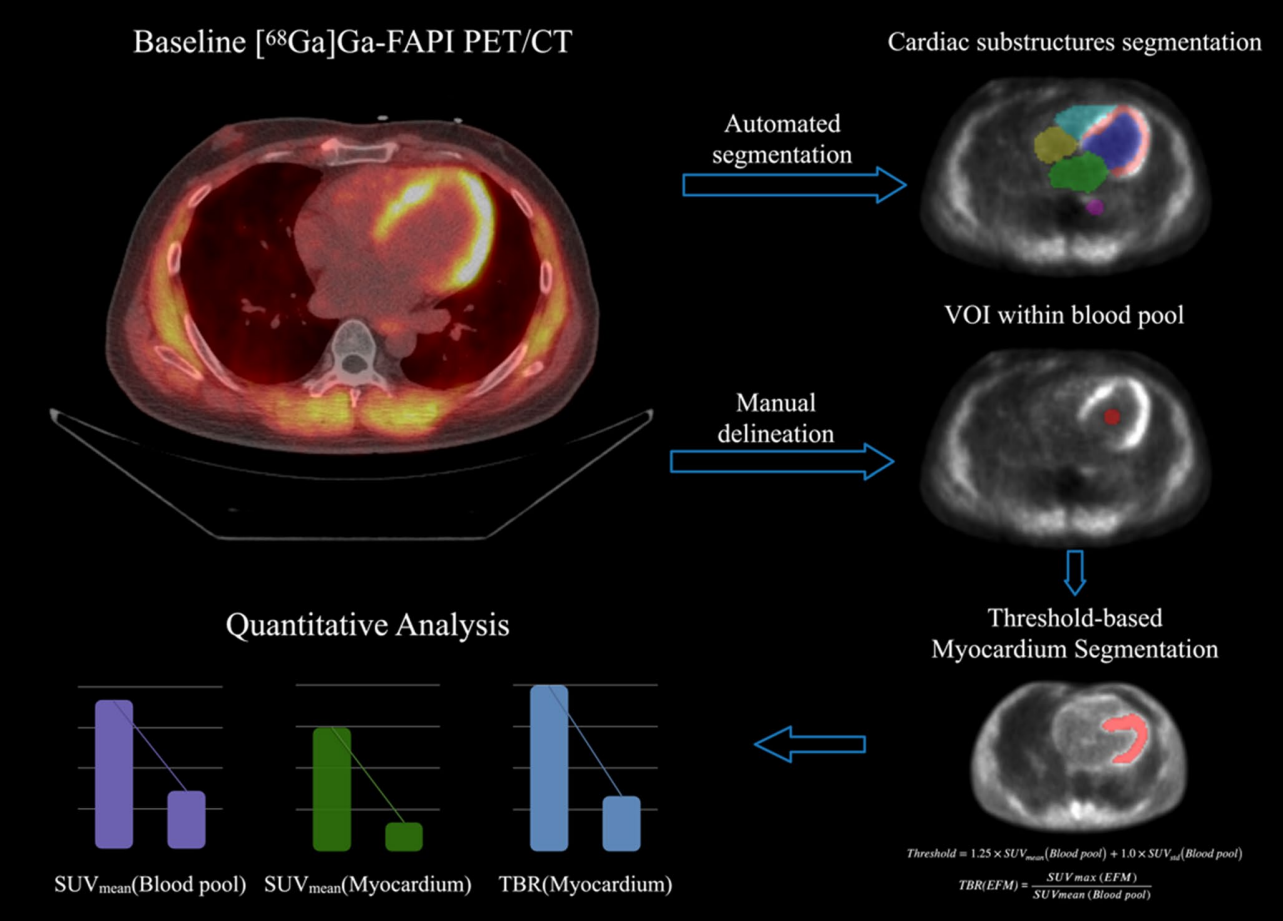
Workflow for myocardial [^68^Ga]Ga-FAPI imaging quantification

As illustrated in Fig. 1, the LV myocardium was segmented on CT using TotalSegmentator [12] and resampled to PET space. A spherical volume of interest (VOI) was placed in the LV blood pool. Standardized uptake values (SUVs) were computed for all VOIs. We define SUV_mean_ (Blood pool) as the mean voxel SUV within this ROI and SUV_std_(Blood pool) as the corresponding standard deviation across voxels. To identify regions of elevated fibroblast activity within the myocardium (EFM), a time point–specific blood-pool–anchored background threshold was defined as:

$$\:\begin{array}{rr}Threshold=&\:1.25\mathrm{*}SU{V}_{\mathrm{mean}}\left(\text{Blood pool}\right)+1.0*SU{V}_{\mathrm{std}}\left(\text{Blood pool}\right)\\\:&\:\end{array}$$

Myocardial voxels with SUV > Threshold were classified as EFM. For each time point, we quantified SUV_mean_(EFM), and the myocardium-to-blood ratio TBR(EFM), defined as:

$$\:TBR\left(\mathrm{E}\mathrm{F}\mathrm{M}\right)=\frac{SU{V}_{\mathrm{m}\mathrm{a}\mathrm{x}}\left(\mathrm{E}\mathrm{F}\mathrm{M}\right)}{SU{V}_{\mathrm{m}\mathrm{e}\mathrm{a}\mathrm{n}}\left(\text{Blood pool}\right)}$$

The three PET time points for each patient were co-registered. The 70- and 120-minute CT images were rigidly aligned to the 60-minute CT, and the resulting transformations were applied to the corresponding PET images. The blood-pool VOI was delineated at 60 min and propagated to 70 and 120 min, with the SUV_mean_ (Blood pool) recomputed at each time to derive time point–specific thresholds and EFM masks.

### Clinical variables and follow-up

Baseline N-terminal pro-brain natriuretic peptide (NT-proBNP, pg/mL) and high-sensitivity C-reactive protein (hsCRP, mg/L) were measured within one week of PET/CT as part of routine pre-TAVI evaluation. Echocardiographic LV ejection fraction (LVEF) and New York Heart Association (NYHA) class were recorded.

At 12 ± 0.8 months after TAVI, follow-up data were available for 11 patients. Because there is no single universally accepted definition of “responder” after TAVI, we prospectively pre-specified a composite responder endpoint to capture clinically meaningful improvement across multiple domains and to minimize misclassification driven by variability of any single measure, particularly in a small cohort. A composite responder status required improvement in ≥ 2 of the following criteria: (a) NT-proBNP decrease ≥ 30% [13]; (b) ≥ 1-class NYHA improvement; (c) hsCRP decrease ≥ 15% if elevated; (d) patient-reported dyspnea “much better”, corresponding to an improvement of approximately 10–15 points on the Kansas City Cardiomyopathy Questionnaire (KCCQ); and (e) LVEF increase ≥ 5% points if baseline LVEF < 60% [14]. All others were classified as non-responders.

### Statistical analysis

Continuous variables are reported as mean ± SD or median (interquartile range [IQR]) as appropriate. Between-group differences were assessed using two-sided Student t tests or Mann–Whitney U tests, and paired data using paired t tests or Wilcoxon signed-rank tests. Comparisons involving more than two groups or repeated measures were analyzed using one-way or repeated-measures ANOVA when assumptions were met, or linear mixed-effects models with subject-level random intercepts when not. Multiple comparisons were adjusted using Tukey or Bonferroni corrections as appropriate. Correlations were evaluated with Pearson or Spearman coefficients and ordinary least squares regression, and change-from-baseline effects were assessed using ANCOVA. All tests were two-sided, with statistical significance defined as *p* < 0.05. Statistical analyses were performed in Python (version 3.10).

## Results

### Patient population

Nineteen patients were included (mean age 78.7 ± 3.6 years; 4 of 19 [21.1%] men; median BMI 28.7 kg/m² (21.5–41.2). Hypertension was present in 16 of 19 (84.2%), diabetes in 7 of 19 (36.8%), and prior stroke in 3 of 19 (15.8%). Median NT-proBNP was 1,061 pg/mL (127–4,827), and median hsCRP was 2.7 mg/L (0.4–49.4). Median LVEF was 60% (34%–82%) and median LVEDV was 118 mL (68–239). Baseline medications included statins (17/19 [89.5%]), ACE-inhibitors/ARBs (15/19 [78.9%]), β-blockers (13/19 [68.4%]), aspirin (7/19 [36.8%]), and calcium-channel blockers (5/19 [26.3%]) ( Table 1).

**Table 1:** Baseline characteristics of the study cohort

Age (y)	78.7±3.6
Sex (n)	
Male	4 /19 (21.1%)
Female	15 /19 (78.9%)
BMI, kg/m²	28.67 (21.5-41.2)
Comorbidity, n/N (%)	
Diabetes mellitus	7/19 (36.8%)
Hypertension	16/19 (84.2%)
Stroke	3/19 (15.8%)
Laboratory	
NT-proBNP, pg/mL	1060.5(127-4827)
hsCRP, mg/dL	0.27 (0.04-4.94)
Cardiac Function	
LVEF, %	60% (34%–82%)
LVEDV, mL	117.68 (68–239)
Medication, n/N (%)	
Aspirin	7 (36.8%)
β-Blocker	13 (68.4%)
ACE-Inhibitor/ARB	15 (78.9%)
Statin	17 (89.5%)
Calcium Channel Blockers	5 (26.3%)
PET (Myocardium)	
SUV_mean_ (myocardium)	2.2±0.7
TBR(EFM)*	3.8±0.8
Cutoff*	2.0±0.8
Organ [^68^Ga]Ga-FAPI signal (SUV_mean_)	
Spleen	1.84±0.42
Kidney	2.77±0.5
Liver	2.13±0.42
Adrenal glands	1.59±0.31
Lung	0.99±0.41
Bone marrow	1.12±0.26
Aorta	2.53±0.56
Pulmonary vein	2.28±0.47

Data are presented as n/N (%) for categorical variables and mean ± standard deviation or median (interquartile range) for continuous variables, as appropriate. Percentages are accompanied by numerators and denominators. SI units are used throughout.

*ACE* angiotensin-converting enzyme, *ARB* angiotensin receptor blocker, *BMI* body mass index, *FAPI* Fibroblast activation protein inhibitor, *hsCRP* high sensitivity C-reactive protein, *IQR* interquartile range, *LVEDV* left ventricular end-diastolic volume, *LVEF* left ventricular ejection fraction, *NT-proBNP* N-terminal pro–B-type natriuretic peptide, *PET/CT* positron emission tomography/computed tomography, *SUV* standardized uptake value, SUV_mean_ mean standardized uptake value, SUV_max_ maximum standardized uptake value, TBR(EFM) SUV_max_ (myocardium) / SUV_mean_ (blood pool), Cutoff SUV_mean_ (blood pool) + 1 *SD

## Myocardial [^68^Ga]Ga-FAPI uptake across time points

**Fig. 2:**
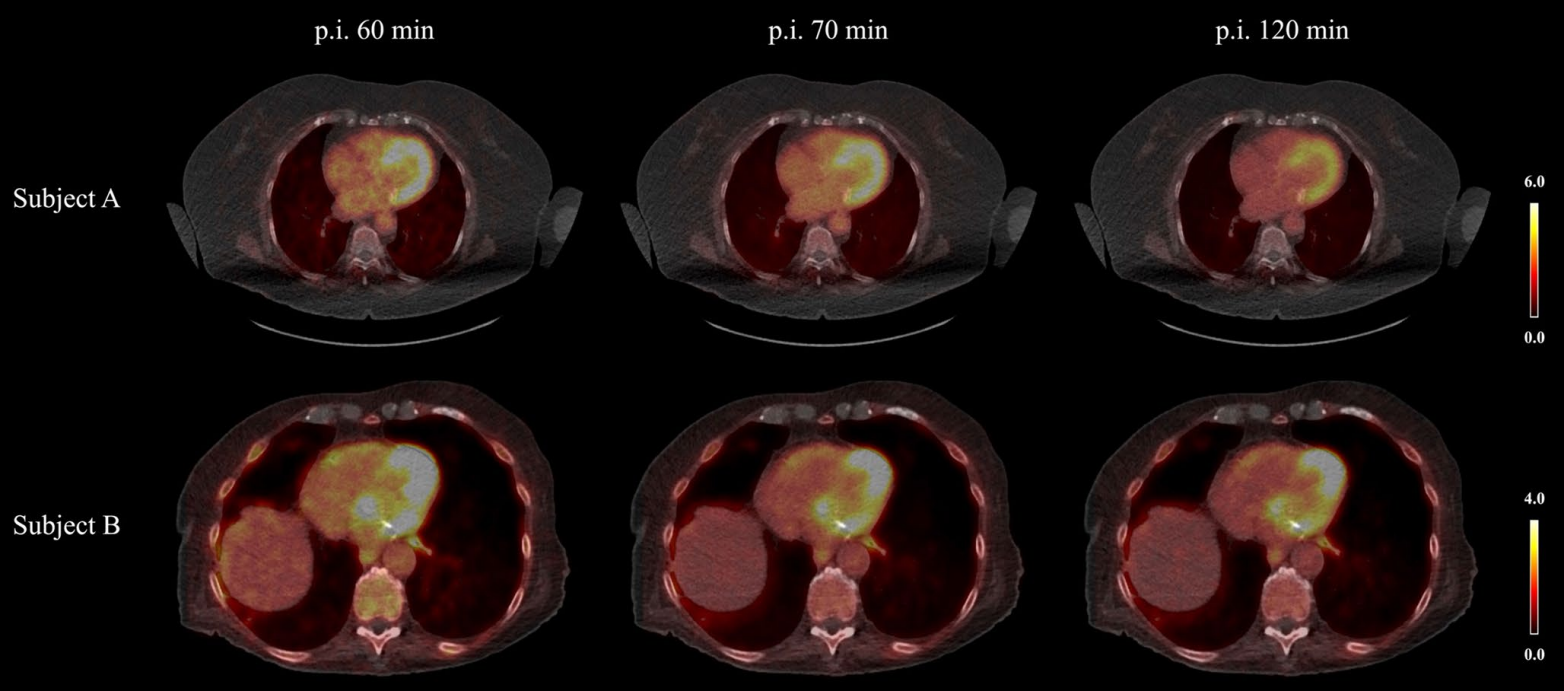
Visual analysis

All patients completed PET/CT imaging at 60 and 70 min p.i., 17 out of 19 patients also underwent the 120-minute acquisition. Visual inspection revealed heterogeneous myocardial uptake patterns, typically more pronounced in the basal and mid-ventricular segments and along the septum (Fig. 2). Blood pool activity declined steadily across time points, whereas myocardial uptake decreased to a lesser extent, thereby improving myocardial-to-blood contrast at later times.

**Fig. 3:**
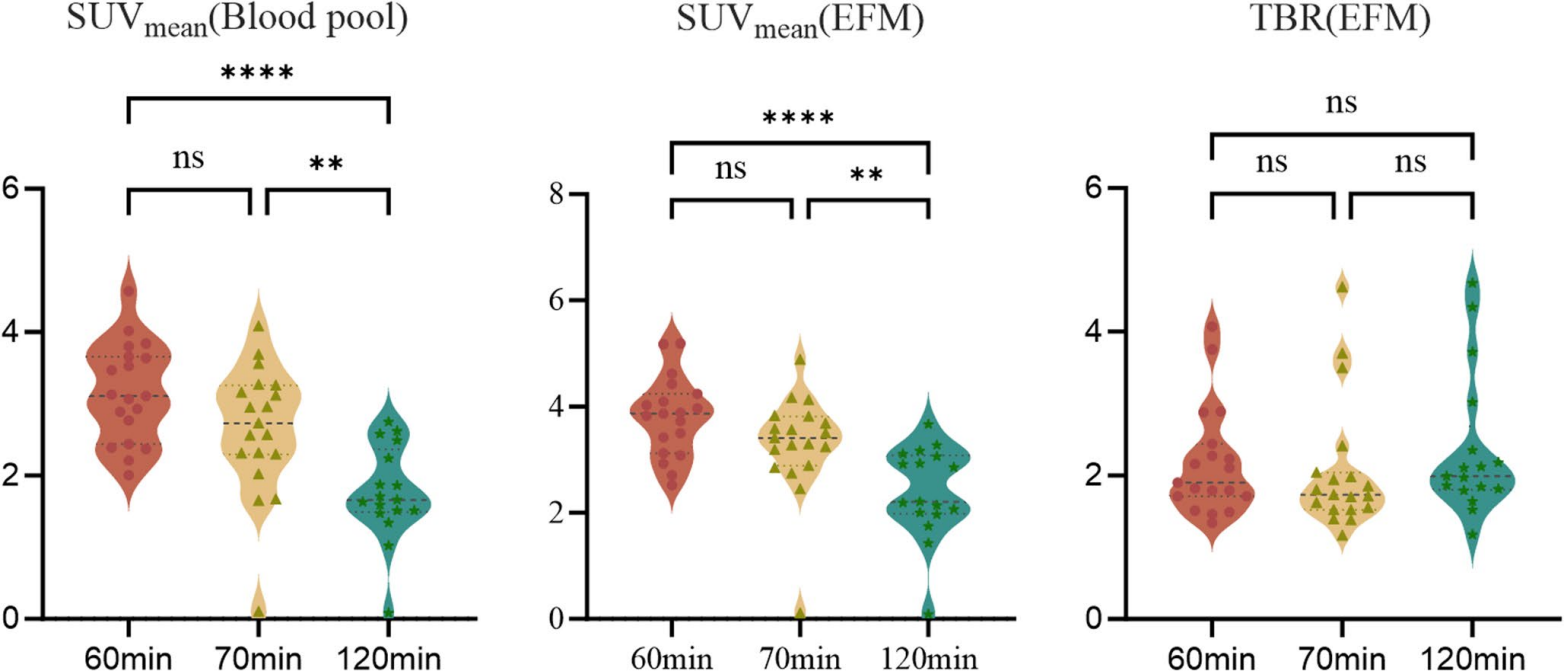
Myocardial and blood pool [^68^Ga]Ga-FAPI uptake over time. Violin plots show distributions with individual data points at 60, 70 (*n* = 19 each), and 120 min (*n* = 17). Brackets indicate pairwise post-hoc comparisons after repeated-measures testing; ns = not significant, **p < 0.01, ****p < 0.0001

Quantitative analysis in Fig. 3 confirmed that, myocardial SUV_mean_ (EFM) was 3.8 ± 0.7 at 60 min and 3.3 ± 1.0 at 70 min (*p* = 0.168), but declined significantly to 2.1 ± 0.9 at 120 min (*p* < 0.001 vs. both 60 and 70 min). In contrast, TBR(EFM) remained stable across time points (2.2 ± 0.8 at 60 min, 2.1 ± 0.9 at 70 min, 2.3 ± 0.9 at 120 min; ANOVA *p* = 0.596), indicating proportional clearance of tracer from both myocardium and blood pool. SUV_mean_ (Blood pool) showed the steepest decline (3.2 at 60 min to 1.8 at 120 min on average), consistent with systemic tracer clearance. Together, these results demonstrate that while absolute myocardial activity diminishes over time, the myocardium-to-blood ratio remains constant beyond 60 min.

### Associations with NT-proBNP and LVEF

**Fig. 4:**
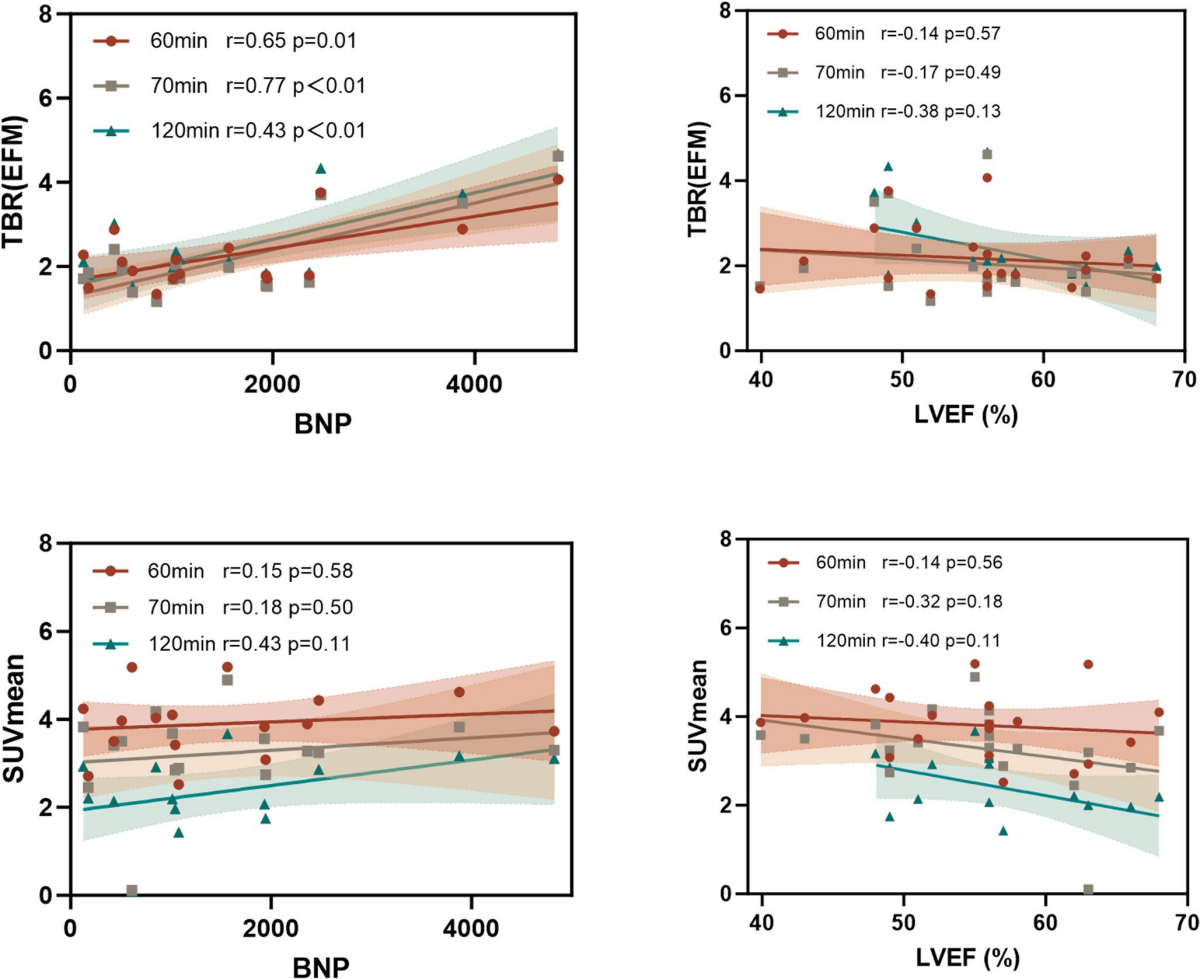
Association of myocardial [⁶⁸Ga]Ga-FAPI uptake with BNP and LVEF. Scatterplots with linear regression fits (shaded 95% CI) depict the relationships between BNP and TBR(EFM) / SUV_mean_, and between LVEF (%) and TBR(EFM) / SUV_mean_. Symbols/colors indicate 60-, 70-, and 120-min post-injection acquisition time points; corresponding r and p values are displayed in each panel

Baseline NT-proBNP ranged from 127 to 4,827 pg/mL (median 1,060.5 pg/mL), reflecting heterogeneity in heart-failure severity. As demonstrated in Fig. 4, baseline TBR(EFM) correlated positively with NT-proBNP at all time points (60 min: *r* = 0.65, *p* = 0.007; 70 min: *r* = 0.77, *p* = 0.005; 120 min: *r* = 0.72, *p* = 0.003). In contrast, SUV_mean_ (EFM) was not significantly associated with NT-proBNP. Across time points, both TBR(EFM) and SUV_mean_ (EFM) exhibited weak inverse trend with LVEF. Collectively, these findings indicate that TBR(EFM) reflects hemodynamic stress–related fibroblast activity more consistently than raw uptake, whereas relationships with systolic function were not demonstrable in this cohort.

### Baseline [^68^Ga]Ga-FAPI uptake and 1-Year outcomes

Of the 19 patients, 11 had complete 1-year follow-up after TAVI. Responders (*n* = 5), defined by improvement in at least two clinical domains, showed a mean 55% reduction in NT-proBNP, improvement by 1–2 NYHA classes, and reduced symptoms and hsCRP. Non-responders (*n* = 6) had minimal change or worsening in NT-proBNP (two patients increased), persistent NYHA limitations, and/or elevated hsCRP despite technically successful valve relief on echocardiography.

**Fig. 5:**
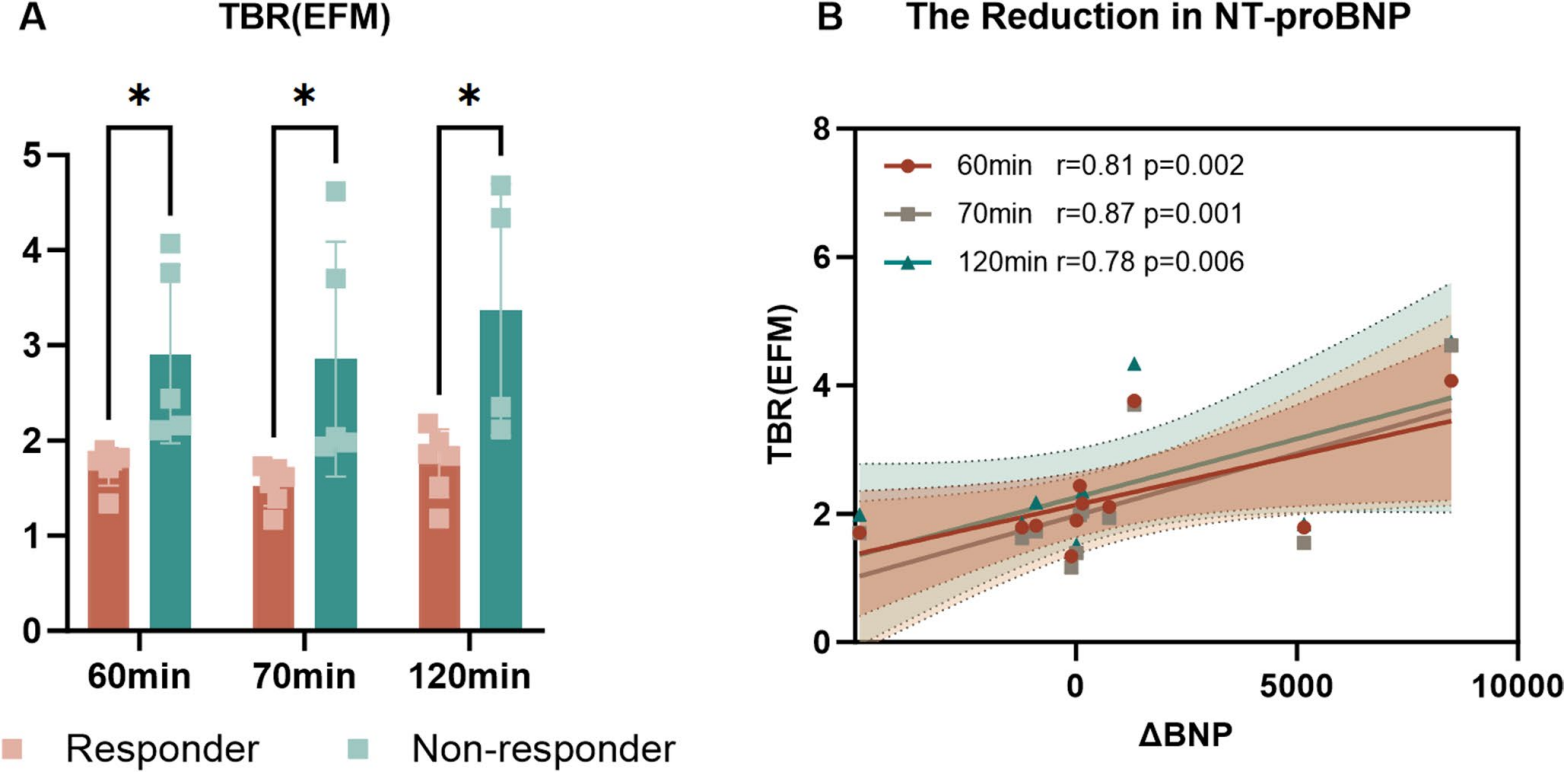
Baseline myocardial ${TBR}\left(EMF\right)$ and 1-year outcomes after TAVI. A: Group comparison of TBR(EFM) in responders vs. non-responders at 60, 70, and 120 min (Mann–Whitney tests; *p < 0.05, **p < 0.01, ***p < 0.001). B: Relationship between baseline TBR(EFM) and change in NT-proBNP at 1 year; Linear regression lines with shaded 95% CI are shown for each acquisition time point, with corresponding r and p values indicated

Baseline [^68^Ga]Ga-FAPI uptake differed markedly by outcome. Responders had lower myocardial TBR(EFM) at 60 min (1.7 ± 0.2) than non-responders (2.9 ± 0.9; Mann–Whitney *p* = 0.013), separation persisted at 70 min (1.5 ± 0.2 vs. 2.9 ± 1.1, *p* = 0.013) and 120 min (1.7 ± 0.4 vs. 3.4 ± 1.3, *p* = 0.019), shown in Fig. 5A. Additionally, while a 60-minute TBR(EFM) cutoff of approximately 2.0 achieved complete separation between responders and non-responders, this post-hoc finding should be considered hypothesis-generating rather than a definitive clinical decision threshold. In contrast, no difference was observed in SUV_mean_ at all time-points (*p* > 0.05), shown in Supplementary Fig. 3. As demonstrated in Fig. 5B, baseline TBR(EFM) significantly correlated with the 1-year change in NT-proBNP derived from all time-points (60 min: *r* = 0.81, *p* = 0.002; 70 min: *r* = 0.87, *p* = 0.001; 120 min: *r* = 0.78, *p* = 0.006), indicating that higher initial [^68^Ga]Ga-FAPI uptake associated with less reduction in NT-proBNP after TAVI. While baseline SUV_mean_ showed no correlation (*p* > 0.05), shown in Supplementary Fig. 3.

## Discussion

This study in patients with severe aortic stenosis undergoing TAVI yielded three principal findings with important implications for imaging protocol design for FAPI uptake, pathophysiological understanding, and patient management. First, the TBR(EFM) was markedly stable between 60- and 120-minutes p.i., even as absolute myocardial SUV declined over the same interval. Second, TBR(EFM) showed robust associations with NT-proBNP across imaging times, indicating that ratio-based quantification better reflects the biology of fibroblast activation under pressure overload. Third, higher baseline (prior to TAVI) TBR(EFM) was associated with limited clinical improvement one year after TAVI, suggesting that pre-procedural [^68^Ga]Ga-FAPI uptake predisposes the feasibility of reverse remodeling and has a significant prognostic value. Collectively, these findings support a streamlined and standardized workflow for myocardial [^68^Ga]Ga-FAPI-PET quantification in AS, consisting of a single static acquisition approximately 60 min post-injection, combined with LV masking and a central blood-pool reference.

The time stability of TBR(EFM) after approximately 60 min p.i. is consistent with rapid blood clearance and early, specific binding of [^68^Ga]Ga-FAPI tracers to activated fibroblasts, yielding proportional washout from myocardium and blood pool beyond the first hour. This behavior supports standardizing acquisition at a single early time point—60 min p.i.—without sacrificing quantitative fidelity. In contrast, the observed 25–30% decline in SUV_mean_ between 60 and 120 min underscores the time dependency of absolute uptake [15] and cautions against inter-patient or inter-study comparisons based on raw SUVs when uptake times differ, even modestly. Although acceptable image quality may be achievable somewhat earlier given the fast kinetics of [^68^Ga]Ga-FAPI, a 60-minute standard provides a pragmatic balance between contrast and harmonization.

When interpreted in this context, the superiority of TBR(EFM) over SUV_mean_(EFM) is mechanistically intuitive. By normalizing to blood-pool activity, TBR(EFM) functions as an internal control that mitigates variability in delivery, clearance, and sampling time, thereby isolating a signal that more faithfully reflects fibroblast biology. The tight correlation between TBR(EFM) and NT-proBNP—an established marker of myocardial wall stress and heart-failure severity—supports construct validity, whereas the absence of a meaningful relationship between SUV_mean_ and NT-proBNP illustrates how absolute measures can obscure clinically relevant associations [16]. Using whole-LV segmentation, elevated TBR(EFM) values substantially above unity indicates global pathological fibroblast activation; volume-based “fibrosis burden” metrics can complement this by localizing disease but require thresholding, whereas TBR(EFM) provides a threshold-free, continuous gauge of global activity.

A practical strength of this work is the self-developed, semi-automatic threshold-based segmentation and quantification pipeline used for myocardial analysis. By combining consistent anatomical masking with reproducible, threshold-driven measurements, the approach reduces operator dependence, shortens processing time, and facilitates quality control. Importantly, it aligns naturally with TBR(EFM)-based reporting, making the method easy to implement at scale and well suited for multi-center harmonization and longitudinal studies where standardization and throughput are essential.

The prognostic signal of baseline TBR(EFM) integrates naturally with these quantitative advantages. Patients exhibiting higher pre-TAVI TBR(EFM) demonstrated a lower likelihood of symptomatic or biochemical improvement at one year, supporting the concept that once diffuse, active myocardial remodeling is established, functional recovery following afterload reduction is constrained. Reports of short-term improvements in LVEF after valve intervention in some high-uptake cases can be reconciled by recognizing that acute contractile reserve does not necessarily translate into durable relief from diastolic dysfunction or neurohormonal activation. Clinically, pre-TAVI TBR(EFM) may guide patient counseling, inform the intensity of post-procedural monitoring, and support consideration of adjunctive therapies targeting myocardial fibrosis or heart failure. Recent studies in high-risk post-TAVI patients have shown that sodium–glucose cotransporter 2 (SGLT2) inhibitors, such as dapagliflozin, significantly reduce all-cause mortality and heart failure events compared with standard care [17]. Whether these benefits are linked to changes in myocardial FAPI uptake and modulation of fibrosis after TAVI remains unclear and warrants further investigation. Beyond baseline risk stratification, serial [^68^Ga]Ga-FAPI PET likely be a marker for both persistent adverse and reverse remodeling.

Our observations demonstrate that myocardial FAPI-PET may provide complementary information to established imaging modalities. Cardiac magnetic resonance (CMR) with LGE images and T1/ECV mapping quantifies established collagen crosslinking, deposition, and replacement fibrosis, which are structural hallmarks that may lag behind biological activity, whereas [⁶⁸Ga]Ga-FAPI PET highlights ongoing fibroblast activation and pro-fibrotic signaling [18], providing a more immediate measure of fibrogenic activity, as demonstrated in recent translational studies of cardiac injury (our MS : https://pubmed.ncbi.nlm.nih.gov/40430477/) Echocardiographic strain provides a functional readout that is sensitive to loading conditions; [^68^Ga]Ga-FAPI offers a molecular readout less affected by hemodynamics at the imaging time. A combined approach may help distinguish active from fixed fibrosis and refine the timing of intervention.

The underlying tracer kinetics help explain both the time stability and the prognostic value of TBR(EFM). [^68^Ga]Ga-FAPI tracers bind to fibroblast activation protein with high specificity; by about one-hour p.i., circulating tracer is low, and tissue signal largely reflects bound or retained tracer, with subsequent proportional clearance from tissue and blood. In hearts with high densities of activated fibroblasts, sustained signaling and matrix deposition create a substrate that is stiffer and less likely to reverse, aligning the molecular readout with clinical trajectories. This framework reinforces the importance of timely intervention in AS and positions [^68^Ga]Ga-FAPI PET as a means to estimate where an individual lies on the spectrum from reversible to entrenched remodeling.

Several limitations temper these conclusions. The sample size—particularly for one-year outcomes (*n* = 11)—limits power and precludes multivariable modeling, so results should be considered hypothesis-generating. Two patients lacked 120-minute scans, modestly reducing precision for time-point comparisons. The composite responder definition, while clinically meaningful, includes subjective elements; harder endpoints were too infrequent to analyze. Histopathologic validation was not feasible. Absence of uniform valve hemodynamic data at follow-up may confound associations between FAPI signal and clinical recovery. Future work should prospectively standardize acquisition at 60 min p.i., report TBR(EFM) as the primary quantitative metric, and predefine clinically actionable cut-points (e.g., ROC-guided thresholds). Multicenter studies powered for incremental prognostic testing over NT-proBNP, LVEF, strain, and CMR fibrosis markers are needed to establish generalizability. Longitudinal imaging before and after TAVI can determine whether dynamic changes in TBR(EFM) track remodeling and outcomes, and interventional trials should assess whether therapies that reduce TBR(EFM) improve clinical endpoints. If confirmed, [^68^Ga]Ga-FAPI PET would serve not only as a risk-stratification tool but also as a pharmacodynamics biomarker for antifibrotic strategies in AS.

## Conclusion

In severe AS, molecular imaging of active fibrosis with [^68^Ga]Ga-FAPI PET/CT provides information beyond conventional structural or functional assessments. A blood-pool–normalized myocardial [^68^Ga]Ga-FAPI PET biomarker offers a time-robust readout of fibroblast activity that aligns with hemodynamic stress, and a single time-point acquisition may be sufficient for quantification. In this exploratory cohort, higher pre-TAVI myocardial TBR identified patients at risk of limited clinical improvement, whereas lower values were associated with symptomatic and functional recovery at 1 year, reflecting varying degrees of fibrotic remodeling. These findings are exploratory and hypothesis-generating, rather than confirmatory. Larger, prospective multicenter studies should predefine analytic thresholds, assess reproducibility, and test incremental prognostic value over NT-proBNP, LVEF, and CMR. If confirmed, FAPI-based TBR(EFM) could support more precise patient selection, counseling, and tailored post-TAVI follow-up.

## Supplementary Information

Below is the link to the electronic supplementary material.

Supplementary Material 1

## Data Availability

Due to ethical restrictions related to the consent given by subjects at the time of study commencement, our data sets are available from the corresponding author on reasonable request after permission from the institutional review board of Medical University of Vienna.
