# Supplementary material for: Time-robust myocardial [68Ga]Ga-FAPI PET biomarker reflects aortic stenosis severity and predicts post-TAVI outcomes
Source: Eur J Nucl Med Mol Imaging. 2026 Feb 16;53(6):4078–87. doi: 10.1007/s00259-026-07815-4 (PMC13121330; doi:10.1007/s00259-026-07815-4)
Supplement: Supplementary file 1 — Supplementary Material 1 [file 259_2026_7815_MOESM1_ESM.docx]

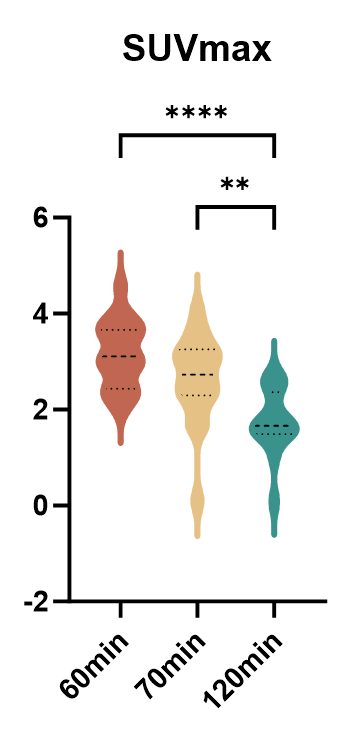


**Supplementary Figure 1.Myocardial and blood pool [^68^Ga]Ga-FAPI uptake over time.** Violin plots show distributions with individual data points at 60-, 70- (n = 19 each), and 120-min (n = 17). Brackets indicate pairwise post-hoc comparisons after repeated-measures testing; ns = not significant, **p < 0.01, ****p < 0.0001.


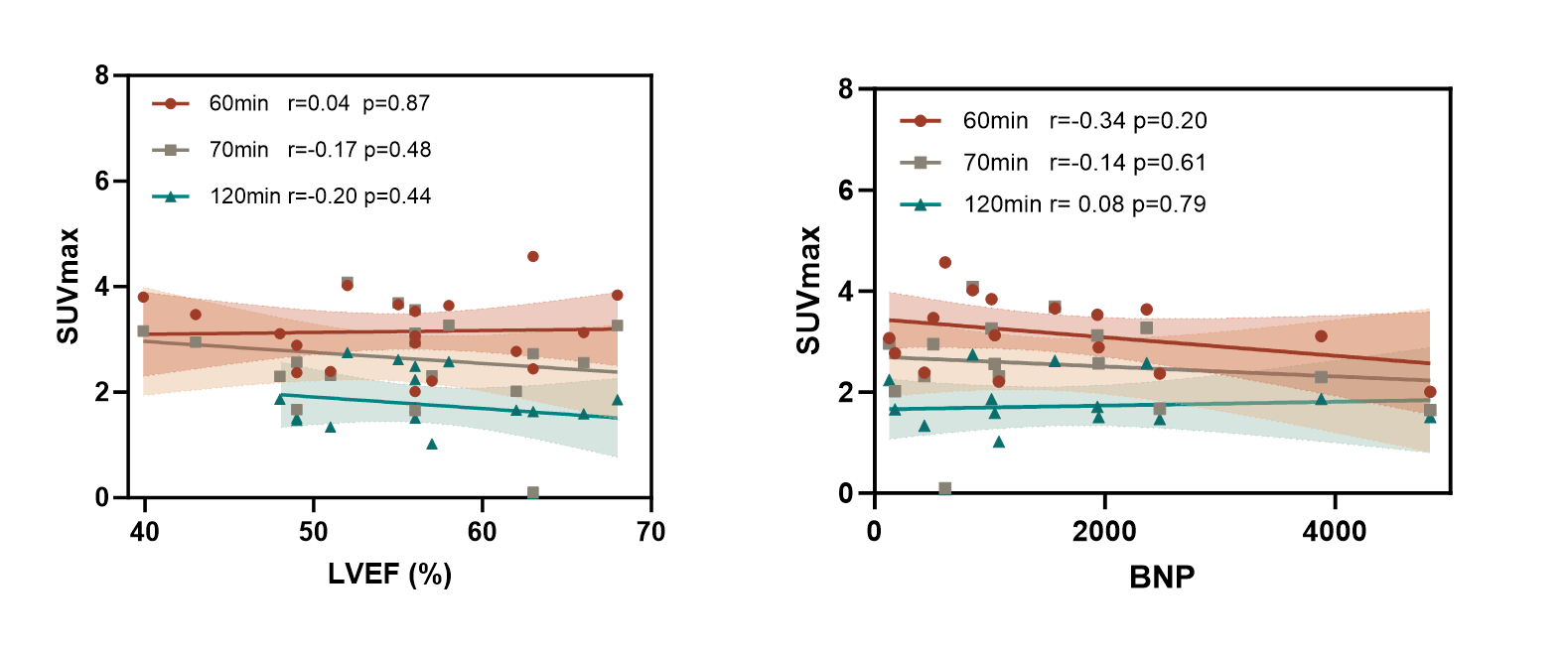


**Supplementary Figure 2. Association of myocardial [^68^Ga]Ga-FAPI uptake with NT-proBNP and LVEF.** Scatterplots with linear fits (shaded 95% CI) depict the relationships between BNP and SUV_max_(EFM), and between LVEF and SUV_max_(EFM). Symbols/colors denote 60-, 70-, and 120-min acquisition time points; corresponding r and p values are displayed in each panel.


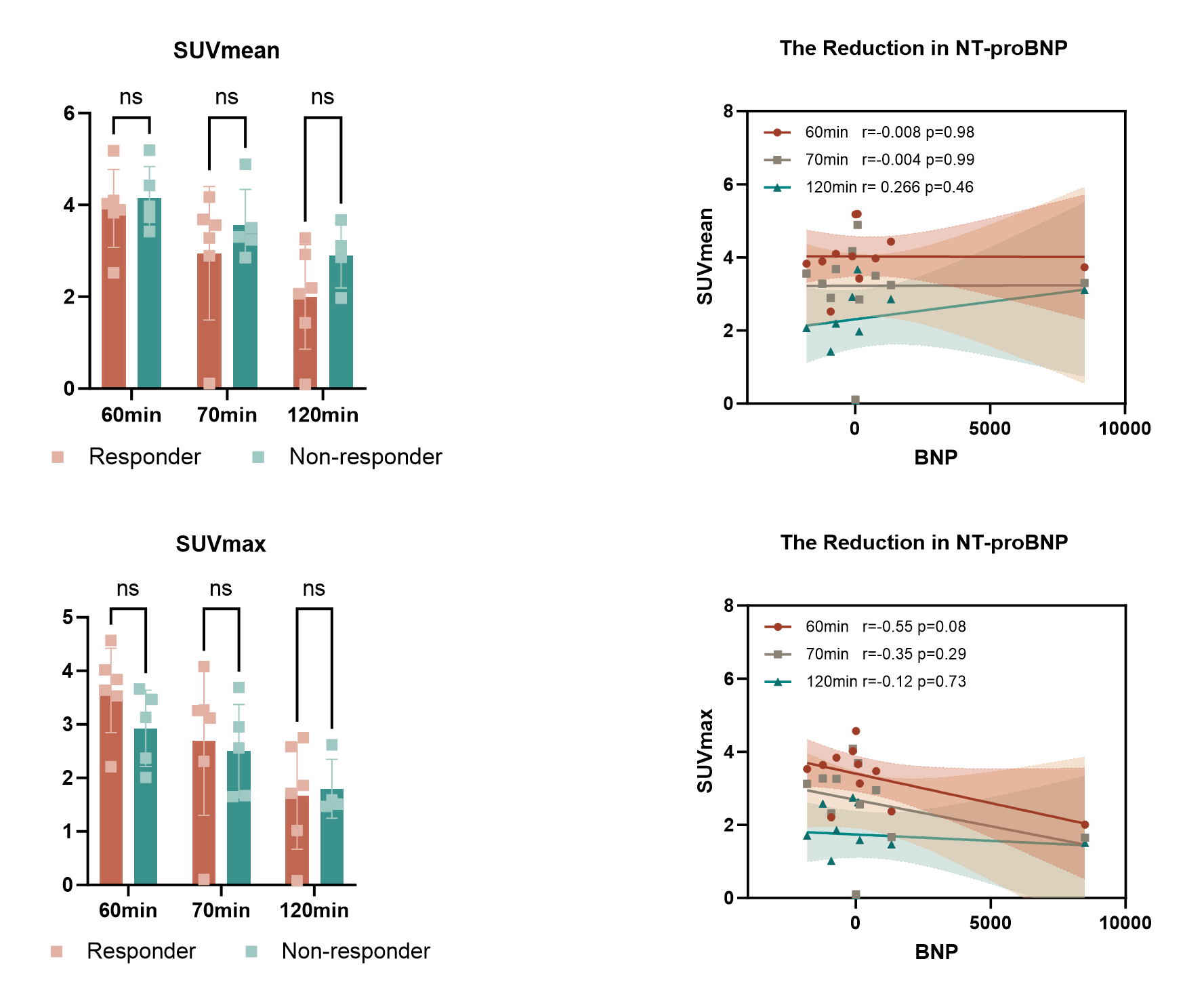


**Supplementary Figure 3.** **Baseline myocardial SUV_mean_ / SUV_max_** **and 1-year outcomes after TAVI.** Group comparison of SUV_mean_(EFM) / SUV_max_(EFM) in responders vs non-responders at 60, 70, and 120 min (Mann–Whitney tests; *p < 0.05). Relationship between baseline SUV_mean_ / SUV_max_ and change in NT-proBNP at 1 year; Linear regression lines with shaded 95% CI are shown for each acquisition time point, with corresponding r and p values indicated.
